# Enhancing cancer therapy *via* acoustics: chemotherapy-enhanced tunable acoustofluidic permeabilization (ChemoTAP)

**DOI:** 10.1039/d5lc00419e

**Published:** 2025-10-21

**Authors:** Ruoyu Zhong, Ke Li, Kaichun Yang, Qian Wu, John D. H. Mai, Joseph Rich, Ying Chen, Xianchen Xu, Jianping Xia, Neil Upreti, Ke Jin, Shujie Yang, Mingyuan Liu, Tony Jun Huang

**Affiliations:** a Thomas Lord Department of Mechanical Engineering and Materials Science, Duke University Durham NC 27708 USA ying.chen3@duke.edu tony.huang@duke.edu; b Alfred E. Mann Department of Biomedical Engineering, University of Southern California Los Angeles CA 90089 USA; c Department of Biomedical Engineering, Duke University Durham NC 27708 USA; d Department of Mechanical Engineering and Applied Mechanics, University of Pennsylvania Philadelphia PA 19104 USA; e Department of Electrical and Computer Engineering, Duke University Durham NC 27708 USA

## Abstract

Mechano-chemo cancer treatment is an emerging therapeutic strategy that enhances chemotherapy efficacy by combining chemical agents with mechanical forces to improve drug uptake and overcome resistance. However, current approaches for delivering mechanical forces, including magnetic stress, hydrodynamic shear, and ultrasonic cavitation, suffer from limited tunability, poor spatial precision, and off-target effects, restricting their clinical potential. Here, we introduce ChemoTAP (chemotherapy-enhanced tunable acoustofluidic permeabilization), an acoustofluidic system that utilizes standing surface acoustic waves (SAWs) to achieve highly localized, tunable mechanical stimulation, enhancing tumor cell permeability and improving chemotherapeutic efficiency. By fine-tuning SAW parameters, ChemoTAP transiently modulates membrane permeability by activating mechanosensitive ion channels, leading to cytoskeletal remodeling and a 2.73-fold increase in intracellular calcium ion flux in HeLa cells. This SAW-induced mechanotransduction response synergistically enhances the cytotoxic effects of cisplatin, increasing tumor cell apoptosis by 1.78-fold through mitochondrial membrane depolarization, reactive oxygen species generation, and endoplasmic reticulum stress pathways. Unlike conventional ultrasound-based cavitation methods, ChemoTAP enables precise, non-invasive mechanical stimulation without requiring microbubbles, offering a controllable and scalable alternative for mechano-chemo cancer treatment. ChemoTAP establishes a foundation for further studies in mechanotherapy treatment pathways and promotes the broader integration of acoustics in oncology.

## Abstract 2

A chemo-acoustic tumor stimulation system amplifies cisplatin-induced apoptosis by elevating ROS levels *via* mechanical modulation.

## Graphical abstract

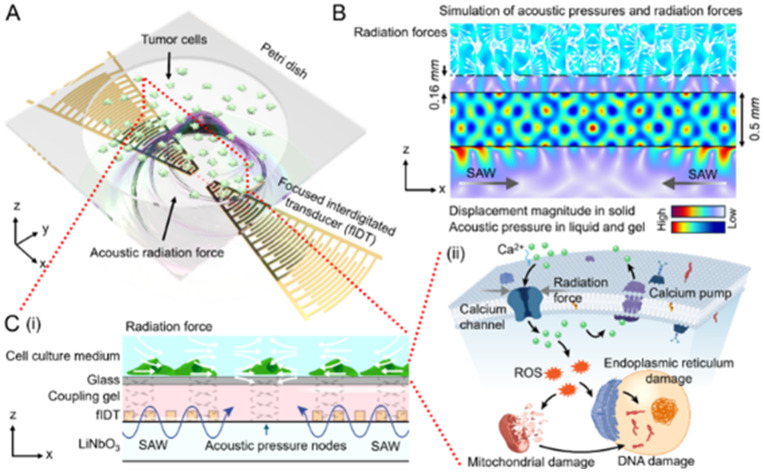

## Introduction

Synergistic mechano-chemo therapy is an emerging paradigm in cancer treatment that enhances drug efficacy by leveraging mechanical stimuli to improve drug uptake, selectivity, and mechanotransduction-based therapeutic responses.^1–6^ This innovative therapy strategy inspires the development of mechanosensitive chemotherapeutic agents, discovery of mechanotransduction pathways, and strategies to overcome drug resistance.^7–9^ By transiently increasing membrane permeability and modulating intracellular signaling,^10,11^ mechanical stimulation facilitates more efficient drug delivery, addressing key limitations of conventional chemotherapy such as low specificity, poor tumor penetration, and acquired resistance.^7–9^

However, currently, the synergistic mechano-chemo therapy remains in the early stages. Despite the great potential, this innovative therapy method faces critical challenges, including mechanical force precise controlling issue,^12–17^ drug-mechanical interactions problem,^18^ and reproducibility evaluations.^19,20^ Addressing these challenges will be essential for translating this promising therapeutic strategy into widespread clinical application.

Recent advancements in mechano-chemo therapy platforms have explored various mechanical stimulation approaches, including magnetic stress,^21–23^ microfluidic-based mechanical forces (*e.g.*, fluid shear, tensile, and viscoelastic forces),^13,24–26^ and ultrasonic cavitation.^27–30^ While these techniques enhanced drug delivery efficiency and improved tumor cell apoptosis, their inherent limitations hindered the application potential. Magnetic stress methods require the addition of magnetic particles to exert mechanical forces onto cells,^1^ which may lead to issues such as off-target accumulation, potential contamination, and difficulties in particle elimination.^16,31,32^ Likewise, microfluidic-based mechanical forces, such as hydrodynamic^13,17,33^ and wall shear stress,^17,34,35^ can transiently disrupt cellular membranes to improve drug uptake, but their effectiveness varies across cancer types, and precise force tuning requires specialized microfluidic setups with high instrumentation costs.^20,36–38^

Ultrasonic cavitation-based techniques, which utilize low-frequency ultrasound (20 kHz–3 MHz) to create inertial cavitating microbubbles, have also been explored as a means of mechanical stimulation.^14,28,39–41^ When these microbubbles collapse near tumor cells, they generate localized forces capable of increasing membrane permeability. However, this mechanism is often unstable, difficult to control, and highly dependent on bubble dynamics,^12,14,15^ leading to variability in cellular responses. Additionally, both non-bubble ultrasound wave forces and collapsing microbubble forces contribute to membrane disruption,^15,42,43^ which can be difficult to fine-tune for controlled drug uptake. Given these limitations, there is a growing demand for a dynamically adjustable, scalable, and clinically compatible mechanical stimulation platform that can precisely regulate mechanotransduction-driven drug uptake while maintaining high spatial and temporal control.

Here, we introduce ChemoTAP (chemotherapy-enhanced tunable acoustofluidic permeabilization), an *in vitro* acoustofluidic mechano-chemo system that enhances the anti-tumor efficacy of small-molecule drugs through precise acoustic wave stimulation. Unlike lower-frequency ultrasound techniques that generate mechanical forces through cavitation-induced microbubble collapse, ChemoTAP operates at a high-frequency (9.63 MHz) surface acoustic wave (SAW) pulse, generated by focused interdigital transducers (fIDTs). This high-frequency solution directly utilizes acoustic radiation forces to modulate cell membranes with nanoscale precision,^44–46^ enhancing membrane permeability and improves drug efficacy.

With no addition of micro-bubbles, the ChemoTAP provides a great tunability, allowing precise adjustments of stimulation intensity, duration, and duty cycles. Through experiment, the proposed ChemoTAP can induce a 1.73-fold increase in calcium ion flux, indicating a high efficiency in enhancing cell membrane permeability. Furthermore, we utilized the ChemoTAP for synergistic mechano-chemo cell killing assay. The efficacy of cisplatin, an representative chemotherapeutic agent, increased by 1.78-fold with the assistance of ChemoTAP. These results indicate that the ChemoTAP is a promising platform for advancing chemo-acoustic cancer treatments, paving the way for further investigations in *in vivo* models and clinical applications.

## Materials and methods

### Working mechanism of the ChemoTAP system

**Fig. 1:**
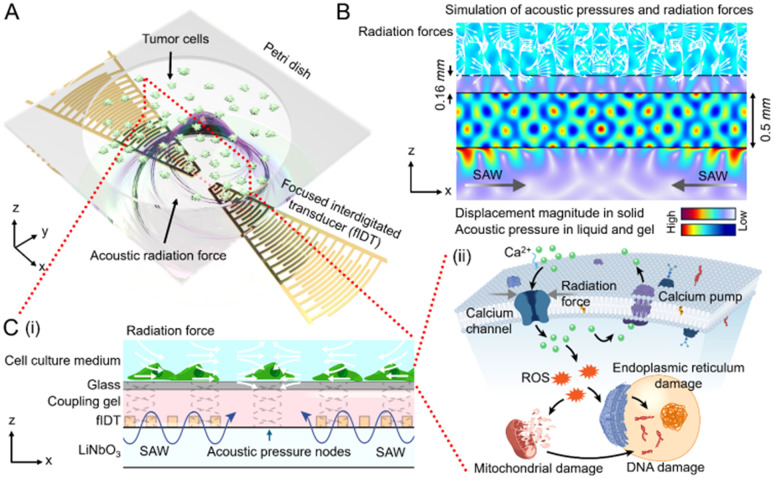
Schematic illustrating the working principle of the ChemoTAP system. (A) Schematic showing a top view of the structure of the ChemoTAP system. The system consists of a separate glass-bottom Petri dish with tumor cells and an attached piezoelectric substrate (LiNbO_3_) equipped with a focused interdigital transducer (fIDT) pair to generate SAWs. (B) Cross-sectional view of a numerical simulation showing the acoustic pressure distribution across the LiNbO_3_ substrate and the Petri dish. (C) Illustration of the acoustic radiation force distribution. (i) Cross-sectional view of the ChemoTAP system. A pair of fIDTs generate standing SAWs, which propagate along the LiNbO_3_ substrate and exert upward radiation forces into the Petri dish, modulating the hydrodynamic environment around the tumor cells and altering their cellular membrane permeability. (ii) In this simplified cellular pathway illustration, ChemoTAP applies an acoustic radiation force to activate calcium ion channels in the HeLa cells, resulting in a calcium ion influx and elevated intracellular ROS levels. This subsequently induces mitochondrial and endoplasmic reticulum damage, ultimately causing DNA disruption and leading to apoptosis in the tumor cells.

The ChemoTAP system (Fig. 1A) applies mechanical forces to adherent cancer cells using standing SAWs generated by a pair of fIDTs. These transducers create a highly localized, energy-concentrated standing wave acoustic field within the focal region between them (Fig. 1B). Operating at a frequency of 9.63 MHz, the acoustic radiation force from the standing wave propagated into the Petri dish and directly acted on the adherent cells by temporarily and reversibly altering their membrane permeability which leads to downstream mechanotransduction responses (Fig. 1C).

**Fig. 2:**
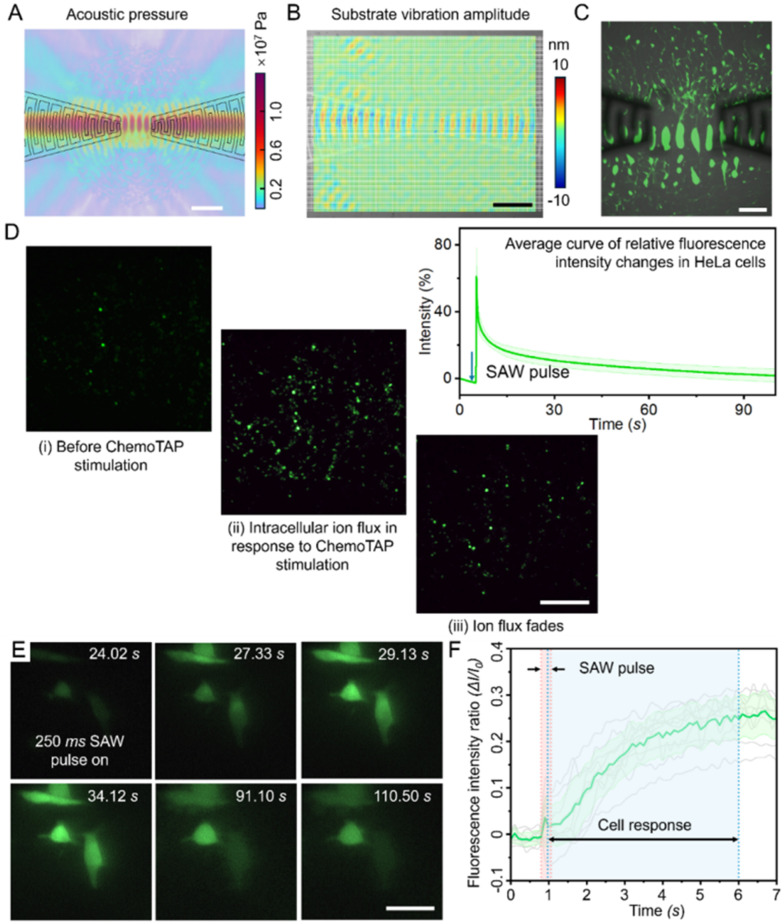
Analytical and experimental findings on ChemoTAP-induced alterations in cell permeability. (A) When a pair of fIDTs are excited at 9.63 MHz, the acoustic pressure nodes are distributed in parallel lines. Scale bar: 1 mm. (B) Vibrometer measurements of substrate displacement show that the area with the largest vibration amplitude corresponds with the numerical simulation results. Scale bar: 1 mm. (C) the pattern of 10 μm green fluorescence beads within the attached Petri dish. The bead distribution aligns well with the acoustic pressure nodes on the substrate. Scale bar: 400 μm. (D) Snapshots display fluo-4 AM-stained HeLa cells, showing significant changes in fluorescence intensity (i) before and (ii and iii) after receiving localized SAW pulse stimulation. The graph of relative fluorescence intensity changes, indicating increased intracellular calcium levels, confirms that SAW pulse stimulation effectively alters cell permeability. Data are graphed as the means ± SD (*n* = 4, biological repeats). Scale bar: 400 μm. (E) Sequential snapshots showing fluorescence intensity changes in four individual cells over a 2 minute period. Scale bar: 20 μm. (F) A representative single-cell response to 250 ms of SAW pulse modulation (*n* = 10).

Previous studies have demonstrated that SAWs can generate both an acoustic streaming drag force and a radiation force.^47–51^ The ChemoTAP system prioritizes enhancing the acoustic radiation force over acoustic streaming to maximize precision. As shown in the 3D numerical simulations using the COMSOL software (Fig. S1), when applying a 9.63 MHz SAW, the acoustic streaming effect in the Petri dish is significantly decreased and the calculated streaming velocities did not exceed 45 μm s^−1^. According to the Stokes drag force equation:

1 *F*_d_ = −6π*ηR*_p_*u*_r_

where *η* is the viscosity of the medium, *R*_p_, and *u*_r_ are the target radius and relative velocity. In this instance, HeLa cells are considered to be the target. The resultant magnitude of *F*_d_ is therefore on the order of 10^−11^ N. In contrast, the equation of acoustic radiation force can be expressed as follows:2
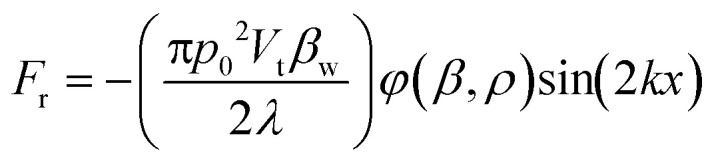
3
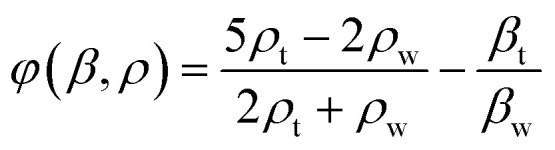
where *p*_0_ and *λ* represnet the acoustic pressure and wavelenght. *V*_t_, *ρ*_t_, and *β*_t_, are the target volume, density and compressibility. *ρ*_w_, and *β*_w_ and density and compressibility of the culture medium, respectively. The acoustic pressure on LiNbO_3_ surface is ∼10^7^ Pa (Fig. 2A), which results in 10^6^ Pa on the Petri dish substrate (Fig. S1). Therefore, HeLa cells would directly experience *F*_r_ on the order of 10^−5^ N, which is approximately 6 orders of magnitude greater than *F*_d_ (Fig. 1B). Thus, the radiation force is dominant, minimizing the effect of acoustic streaming.

In the ChemoTAP design, the curvature of the fIDT fingers (*θ*) enhances the precision of the applied force. Measurements using a vibrometer demonstrate that the energy-focusing design of the fIDT leads to intense localized displacement mostly within the area between the fIDT pair (Fig. 2B). This finding is further validated by particle tracing experiments using 10 μm diameter particles (Fig. 2C). As a result, we can infer that the area between and above the fIDT fingers (∼15.13 mm^2^) is where the cells experience the most intense mechanical stimulation. Theoretical simulations and experimental results confirm the energy-concentrating properties of the fIDT-generated standing SAWs in the ChemoTAP system, which are subsequently utilized for precise acoustic-based mechanical stimulation of cells.

### Device design and fabrication

The ChemoTAP system consists of two key components: a commercial glass-bottom Petri dish (801001, *Φ* 20 mm, Nest Biotech, China) for cultivating adherent cells, and a LiNbO_3_ piezoelectric substrate with fIDTs designed to generate SAWs. In the fIDTs, the electrodes are patterned in a 20° arc around a common focal point. This arrangement ensures that the SAWs generated by the piezoelectric substrate converge at the focal area and maximize the modulation efficiency. The fIDT pair is patterned on a *Y*-128° cut LiNbO_3_ substrate (Precision Micro-Optics, USA) The metal transducer patterning process adheres to standard photolithography protocols, followed by metal deposition of one 5 nm chromium (Cr) adhesion layer and a 150 nm gold (Au) conductive layer *via*. The metal deposition is conducted *via* e-beam evaporation and a subsequent liftoff procedure.^52^

### Experimental operation

Before ChemoTAP stimulation, tumor cells waiting to be modulated are seeded and cultured on the glass-bottom Petri dish. ChemoTAP stimulation experiments were conducted with a medium volume of 200 μL to avoid potential complications with dampening of the acoustic wave and exposing cells to air. The fIDT pair is powered by an amplifier (25A250A, Amplifier Research, USA), which amplifies the signals triggered by an function generator (AFG3102C, Tektronix, USA). Pulsed AC signals will be generated by the function generator, amplified by the amplifier, and converted into acoustic wave pulses. An oscilloscope (InfiniiVision MSOX2024A, Keysight, USA) is used to monitor the pulse voltages that are applied to the fIDT pair. Once all components are properly prepared, before performing SAW modulation, the Petri dish is attached to the fIDT pair using coupling gel (Reed Instruments, USA) to eliminate air gaps and to ensure a tight seal. After the setup is complete, the function generator is turned on to produce a pulse signal for applying SAW modulation to the cells in the Petri dish at a specified intensity and duration. A microscope (TE2000-U, Nikon Ti, Japan) is used for real-time observation, while a digital camera (EOS Rebel T3i, Canon, Japan) records the cellular responses in real-time.

### Cell preparation

A cryopreserved HeLa cell line (CCL-2) was purchased from ATCC company and cultivated in DMEM culture medium (ATCC 30-2003). HeLa cells were cultured under a 5% CO_2_ atmosphere at 37 °C. For subsequent cell experiments, we seeded HeLa cells in glass-bottom Petri dishes, inoculating 1 × 10^5^ cells per dish. For the experimental groups using the Gd^3+^ inhibitor, HeLa cells were pre-treated with 5 mM Gd^3+^ for 5 minutes before starting the ChemoTAP stimulation experiments.

### Characterization of intracellular Ca^2+^ concentration

To characterize changes in the intracellular Ca^2+^ concentration in HeLa cells after different treatments, we employed the Fluo-4 AM calcium indicator for staining. Briefly, once the HeLa cells had fully spread across the Petri dish, they were incubated with Fluo-4 AM at the recommended concentration at 37 °C for 30 minutes, followed by washing with PBS. ChemoTAP stimulation was then applied to the HeLa cells, and the fluorescence changes within the cells were observed using a fluorescence microscope. Additionally, HeLa cells treated under different conditions were collected, and the intracellular fluorescence changes were quantitatively analyzed using flow cytometry.

### Detection of apoptosis levels

To assess the changes in apoptosis levels in HeLa cells caused by cisplatin after ChemoTAP stimulation, we employed flow cytometry. Specifically, we established several experimental groups: control, cisplatin alone, ChemoTAP stimulation alone, ChemoTAP + cisplatin stimulation, and ChemoTAP + cisplatin + Gd^3+^ stimulation. For the groups receiving both cisplatin and ChemoTAP stimulation, cisplatin was first added to the culture medium and, after 12 h, ChemoTAP stimulation was applied. Following the ChemoTAP stimulation, the treated cells were cultured for an additional 12 h. After this culturing period, cell samples were collected and stained according to the steps provided in the Annexin V-FITC apoptosis assay kit. After that, the stained samples were put into the flow cytometry for analysis.

### Changes in mitochondrial membrane potential

To determine whether the Ca^2+^ influx triggered by ChemoTAP stimulation affects the mitochondrial membrane potential in HeLa cells, we used the JC-1 dye to stain the cells after different treatments. To observe changes in mitochondrial membrane potential in real-time, we first stained the cell samples with JC-1. After staining, the cells were washed with PBS. For the groups receiving both cisplatin and ChemoTAP stimulation, cisplatin was first added to the culture medium, and after 12 h, ChemoTAP stimulation was applied. After completing the treatments, we collected cell samples and used flow cytometry to detect changes in mitochondrial membrane potential.

### Detection of apoptosis level immunofluorescence assay for CHOP and RAD51 proteins

To further investigate the effects of ChemoTAP stimulation on the endoplasmic reticulum and DNA repair functions in HeLa cells, we conducted immunofluorescence assays to characterize the expression levels of CHOP and RAD51 proteins. For the groups receiving both cisplatin and ChemoTAP stimulation, cisplatin was first added to the culture medium, and after 12 h, ChemoTAP stimulation was applied. The treated cells were cultured for an additional 12 h after the ChemoTAP stimulation. Following this culturing period, cell samples underwent the immunofluorescence assay steps which included fixation, permeabilization, blocking, incubation with primary antibodies, and incubation with secondary antibodies. The resulting cell samples were stored in the dark and observed under a fluorescence microscope to assess the expression levels of CHOP and RAD51 proteins and their nuclear co-localization.

### Automated fluorescence intensity processing

To statistically analyze dynamic changes in fluorescence intensity across a large number of individual cells, we developed a custom automated MATLAB script (version 2017b). This script tracks and records fluorescence intensity variations over time, generating a table that maps single-cell fluorescence intensity changes against time. The core algorithm follows three main steps: (1) loading the source video of intensity changes, (2) identifying the spatial distribution of cells and assigning each cell a unique identifier (Fig. S2), and (3) tracking fluorescence intensity frame by frame, followed by data collection and export for further analysis. The script accepts video files (*e.g.*, .avi) recorded using a digital camera to capture fluorescence signals within the field of view, and its output is a table where each column represents the fluorescence intensity of an individual cell at different time points. Provided as Note S1, the script requires MATLAB 2017b or later for execution and can be run by executing the main file works.m. The output data were visualized using OriginLab 2018 software (OriginLab Corp., USA) to generate curve plots, violin plots, and ridge plots for further analysis and interpretation.

## Results

### Localized and intense SAWs in the ChemoTAP system reversibly alter cell membrane permeability

To investigate the response of HeLa cells to the designed standing SAWs in the ChemoTAP system, we cultured the cells directly on the disposable Petri dish component of the ChemoTAP system for convenience. The cells were then stained with Fluo-4 AM, and the calcium ion flux was monitored in real-time using a microscope before, during, and after SAW treatment. By tracking the average fluorescence intensity of the cells within the field of view, we observe that our ChemoTAP system robustly activates membrane permeability in the cells (Fig. 2D and Video S1). A significant influx of calcium ions occurs immediately after ChemoTAP modulation, causing the average fluorescence intensity to increase by around 60% and then followed by a return to a baseline intensity level after about 60 s.

Since HeLa cells are adherent, they remain in place after the SAW pulses, allowing for the precise tracking of individual cell responses (Fig. S2). Using high-magnification microscopy and a fast camera, we monitored calcium ion dynamics, response speed, and fluorescent intensity at the single-cell level. This provides detailed data on the effects of SAW modulation (Fig. 2E and F). The four cells shown in Fig. 2E are representative of the single-cell responses in terms of the fluorescent intensity changes and correlate with the flux speed of intracellular calcium. Any variability may be attributed to the cells being positioned differently within the SAW field because the pressure amplitude varies between nodes and antinodes. Additionally, slight differences in the cells' growth states may contribute to variations in the calcium flux response magnitude and speed. We utilized a custom analysis program (running on MATLAB 2017b, MathWorks®, USA) to automatically detect changes in fluorescence intensity in individual cells. It focuses on responses that occur within approximately 8 seconds following SAW modulation (Fig. 2F). When a 250 ms SAW pulse was applied, most cells began responding within a few tens of milliseconds before the pulse ended, as evidenced by a marked increase in intracellular calcium flux intensity. By continuously monitoring the cells, we found that the intracellular calcium as indicated by the fluorescent intensity peaked within ∼5 s, after which the calcium levels began to decline in most cells. These initial results demonstrate the feasibility in inducing a permeability response in mechanosensitive cells using the ChemoTAP platform.

### Optimal ChemoTAP parameters for effective cell stimulation

**Fig. 3:**
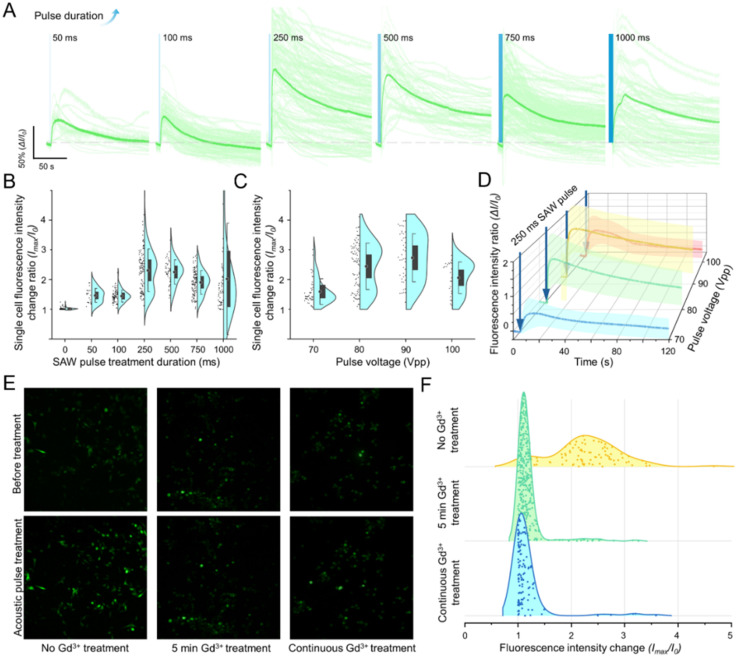
ChemoTAP-based mechanical stimulation mediates cell response *via* mechanosensitive ion channels. (A) Relationship between intracellular calcium ion flux intensity in single HeLa cells and SAW pulse duration. (B) Quantification of HeLa cell response peaks as a function of SAW pulse duration. Data are graphed as the means ± SD (numbers of cells counted in each group: at 0 ms = 86 cells; 50 ms = 20; 100 ms = 83; 250 ms = 114; 500 ms = 45; 750 ms = 121; 1000 ms = 87). (C) Quantification of HeLa cell response peaks as a function of input voltages. (D) Relationship between intracellular calcium ion flux intensity in single HeLa cells with different input voltages. Data are graphed as the means ± SD (numbers of cells counted in each group: at 70 V_pp_ = 47 cells; 80 V_pp_ = 87; 90 V_pp_ = 43; 100 V_pp_ = 25). (E) Images showing the response of HeLa cells with different Gd^3+^ treatments to the same SAW stimulation. Gd^3+^ is a representative mechanosensitive ion channel blocker. Scale bar: 200 μm. (F) Distribution of single HeLa cell response peaks with different Gd^3+^ treatments. In the group without Gd^3+^ treatment, the statistical peak of the fluorescence intensity changes in individual cells is around 2.3. In contrast, in both Gd^3+^-treated groups, the peak remains around 1, indicating no significant intensity change (cell counts for each group: no Gd^3+^ = 88, 5 min Gd^3+^ = 347, continuous Gd^3+^ = 118).

After establishing the feasibility of cell stimulation using our ChemoTAP platform, we then investigated the optimal parameters, including the SAW pulse duration and the pulse amplitude, for maximum cell response. Keeping the input voltage of the SAW pulse constant at 100 V_pp_ to maintain a stable acoustic radiation force, we sequentially increased the duration of the SAW stimulation (Fig. 3A). We found that shorter pulse durations (0–100 ms) failed to fully activate changes in membrane permeability, with the peak fluorescence intensity indicating calcium ion flux was only about half that of the intensity level of the 250 ms pulse duration group. This result could be attributed to the diverse types and multiple numbers of mechanosensitive ion channels within a single cell. When the stimulation duration is too short, some ion channels fail to reach their activation threshold. This results in only partial activation of the cell's ion channels and hence limits the calcium influx.^53,54^ Furthermore, we quantified peak fluorescence intensity changes (*I*_max_/*I*_0_) in all ChemoTAP-stimulated cells within the target region, encompassing both responsive cells exhibiting calcium ion flux and non-responsive cells (defined as those showing no detectable fluorescence changes; Fig. 2B). Statistical analysis revealed that the proportion of non-responsive cells decreased from approximately 67.65% at 50 ms pulse duration to 5.60% at 250 ms. With pulse durations of 250 ms and longer, the average proportion of non-responsive cells was 7.98 ± 2.75% (±standard deviation).

For SAW pulse durations exceeding 500 ms, many cells still exhibited calcium ion flux fluorescence curves similar to those in the 250 ms pulse duration group (Fig. 3A). However, the overall average fluorescent intensity slightly decreased as the SAW pulse duration increased. We attribute this decline to the following factors: 1) the 250 ms pulse duration fully activates membrane permeability, or in other words, mechanosensitive ion channels are opened to their maximum. Beyond this point, the ion channels become saturated, and further stimulation has no additional effect as their permeability has reached its peak. 2) Prolonged treatment time continuously stimulates calcium ion channels, leading to sustained Ca^2+^ influx. This, in turn, activates the cell's self-regulation mechanisms to prevent further Ca^2+^ uptake. Within the cells, there are not only mechanosensitive ion channels that facilitate intracellular Ca^2+^ flux, but also calcium pump channels that actively pump calcium ions out of cells. The presence of calcium pumps is a cellular adaptation to prevent calcium overload.^55^ The cells' protective mechanisms may have been triggered with the longer stimulation pulse ≥750 ms, causing the calcium pumps to activate strongly and pump calcium ions out as calcium flows in. This leads to a stabilization or even a decrease in the overall peak response. Similar phenomena are found in previous studies.^53,54^ Considering these factors, a 250–500 ms duration SAW pulse is the most effective modulation duration for HeLa cells, leading to approximately a 1.31-fold increase in cell permeability.

After determining the optimal SAW pulse duration, we proceeded to optimize the pulse amplitude level of the ChemoTAP system (Fig. 3C and D). The stress level applied to the cell are determined by the voltage applied to the fIDTs. When the input voltage reached 70 V_pp_, a small number of HeLa cells would exhibit a response. Then we further improve the input power, the intensity and proportion of responding cells rapidly increase. To determine the optimal input voltage, we quantified the intracellular calcium ion flux peaks with different input voltages (Fig. 3D). Through comparison, the optimal input condition occurs when the input voltage is set at 90 V_pp_ (pulse duration is set at 250 ms in all groups). Under the optimal conditions, the strongest cellular response is observed, with the peak intracellular calcium ion flux intensity reaching approximately 2.73 times that of the resting state.

An interesting phenomenon is observed in the 100 V_pp_ treatment group where the intensity peak is slightly lower than the 90 V_pp_ treatment group. This effect could be attributed to cellular protective mechanisms as well. Sustained SAW-induced calcium influx could activate calcium pumps and other regulatory channels, helping to balance internal calcium levels. When exposed to SAW stimulation beyond a certain threshold, excessive acoustic pressure may force cells into a state of “fatigue” or desensitization which ultimately reduces their responsiveness. Similar trends have been observed in studies on intracellular delivery efficiency, where the acoustic stimulation power shows a peak effect then after which any further increases in the input power led to diminished results.^54,56^

### SAW stimulation in the ChemoTAP system activates mechanosensitive ion channels

After demonstrating that SAW stimulation in the ChemoTAP system can rapidly and reversibly alter cell permeability, we further investigate whether the mechanical forces generated by the ChemoTAP system are the primary mechanism modulating the mechanosensitive ion channels (Fig. 1C(ii)). This mechanism hypothesis is validated by the experiments shown in Fig. 3E and F. We cultured HeLa cells in three groups under different conditions: no pre-treatment group, a ChemoTAP-stimulated group treated with Gd^3+^, which is a blocker of mechanosensitive ion channels,^57,58^ for 5 minutes, and a ChemoTAP-stimulated group continuously treated with Gd^3+^. After ChemoTAP stimulation, the group without Gd^3+^ treatment exhibits a clear increase in fluorescence intensity across the entire field. In contrast, almost no fluorescence intensity change is observed in the two Gd^3+^-treated groups (Fig. 3E). Fluorescence intensities at the single-cell level are also tracked (Fig. S3 and 3F). These results strongly support the idea that SAW stimulation in the ChemoTAP system directly influences cell permeability by activating mechanosensitive ion channels, resulting in a rapid influx of calcium ions within a short timeframe.

These results allowed us to identify the optimal ChemoTAP stimulation parameters for HeLa cells. With these optimized conditions, we were able to further explore the mechanism and effects of ChemoTAP-induced changes in cell permeability, offering valuable insights for advancing ChemoTAP-enhanced mechano-chemotherapy applications.

### ChemoTAP induces calcium influx, enhancing the efficiency of mechano-chemo cell apoptosis

**Fig. 4:**
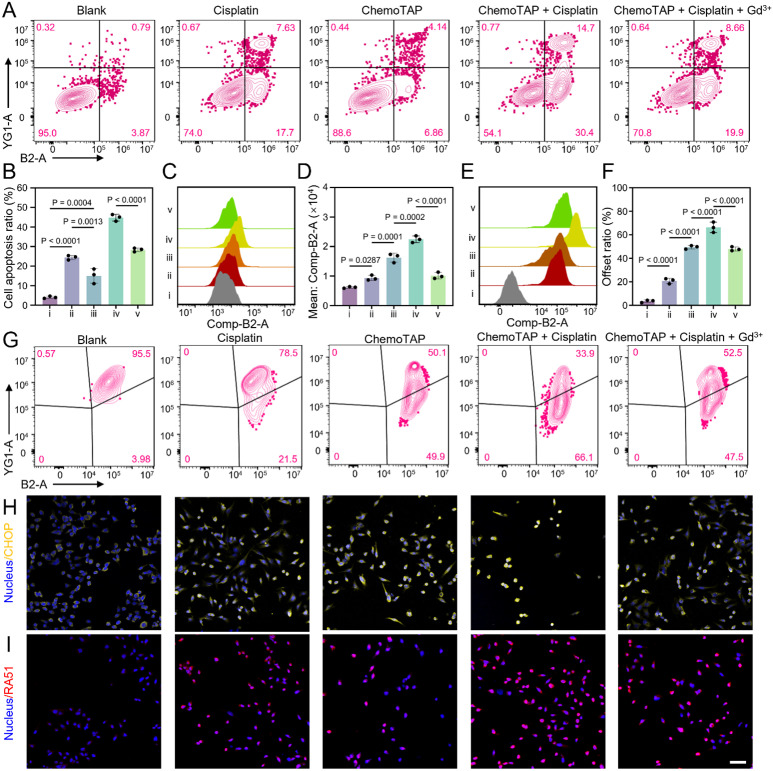
Anti-tumor efficiencies *via* ChemoTAP-based mechano-chemo stimulation. (A) Apoptosis levels in HeLa cells following different treatments. (B) Proportion of apoptotic cells. (C) Intracellular calcium ion concentration in HeLa cells after different treatments. (D) Flow cytometry provides quantitative analysis of intracellular calcium ion fluorescence. (E) Flow cytometry provides quantitative analysis of intracellular ROS level. (F) Statistical analysis of mitochondrial membrane polarization ratio using flow cytometry. Labels in (B–F) represent: i) Blank group; ii) Cisplatin treated group; iii) ChemoTAP treatment group; iv) ChemoTAP and cisplatin cotreatment group; and v) ChemoTAP, cisplatin, and Gd^3+^ cotreatment group. (G) Mitochondrial membrane potential changes in HeLa cells following different treatments. (H) Immunofluorescence analysis of CHOP protein expression in HeLa cells. (I) Immunofluorescence analysis of RAD51 protein expression in HeLa cells following different treatments. Scale bar: 50 μm.

To further validate whether our ChemoTAP system increases the sensitivity of tumor cells to nanomedicines, we selected cisplatin, a drug commonly used in the clinical treatment of cervical cancer, with HeLa cells serving as a model.^59^ Initially, we assessed the apoptotic behavior of HeLa cells within 24 hours under the combined effect of ChemoTAP stimulation and cisplatin. As shown in Fig. 4A and B, the individual effects of ChemoTAP stimulation or cisplatin on killing HeLa cells is relatively low, at 25.33% and 11.00% respectively. However, under the combined use of ChemoTAP stimulation and cisplatin, the apoptosis rate is 45.1%. Note that upon adding the Gd^3+^ inhibitor, the apoptosis rate is significantly decreased and is nearly similar to that of the group treated solely with cisplatin. This result indicates that ChemoTAP stimulation can significantly enhance the chemotherapeutic efficacy of cisplatin on HeLa cells. Subsequently, we further explored the response behavior of HeLa cells after receiving ChemoTAP stimulation. By collecting cells before and after ChemoTAP stimulation and using flow cytometry to detect Ca^2+^ concentration changes inside the HeLa cells (Fig. 4C and D), we found that the intracellular calcium concentration in HeLa cells greatly increased after ChemoTAP stimulation. However, this behavior disappeared with the addition of the Gd^3+^ inhibitor. This observation is consistent with the results shown in Fig. 3E and F. According to previous reports,^60,61^ a large influx of calcium ions can lead to an increase in intracellular reactive oxygen species (ROS) content, which are known to disrupt cellular homeostasis and promote apoptosis,^62^ and cause damage to the mitochondria and endoplasmic reticulum. To verify this behavior, we used flow cytometry to measure the ROS levels in HeLa cells after different treatments. As shown in Fig. 4E, the ROS content inside the cells after ChemoTAP stimulation is significantly richer when comparing to the untreated group (control group). Moreover, the introduction of the Gd^3+^ inhibitor restores ROS levels back to normal.

Additionally, we used the JC-1 probe to characterize the changes in mitochondrial membrane potential in HeLa cells after different treatments. As shown in Fig. 4F and G, ChemoTAP stimulation of HeLa cells leads to changes in the mitochondrial membrane potential, resulting in mitochondrial damage. Subsequently, we further characterized the endoplasmic reticulum damage in the HeLa cells using immunofluorescence assays. As shown in Fig. 4H, following ChemoTAP stimulation, the CHOP protein shows significant activation and co-localization within the nuclei in HeLa cells, indicating typical endoplasmic reticulum stress. The immunofluorescence results for the RAD51 protein also shows significant activation and co-localization with nuclei after ChemoTAP stimulation (Fig. 4I), indicating that ChemoTAP stimulation caused DNA damage in the HeLa cells and triggering DNA repair mechanisms. In summary, ChemoTAP stimulation activates ion channels. This leads to an influx of calcium ions, increases intracellular ROS levels which causes damage to the mitochondria and endoplasmic reticulum, and ultimately enhances the cytotoxic efficacy of cisplatin against HeLa cells (Fig. 4G).

## Conclusion and discussion

This study demonstrates the ChemoTAP (chemotherapy-enhanced tunable acoustofluidic permeabilization) as a powerful synergistic mechano-chemo cancer treatment tool.^63–65^ ChemoTAP shows a strong ability to modulate cell membrane permeability effectively and efficiently. It eliminates the need for additional external components, such as micro-bubbles and magnetic particles. Moreover, the self-contained modular design ensures seamless integration with standard benchtop cell culture, detection, and analysis techniques, making it more accessible and scalable than many existing microfluidic-based mechanical modulation technologies.^66–68^ By significantly simplifying workflow and improving reproducibility,^68–71^ ChemoTAP establishes a robust foundation for further development in acoustofluidic-based precision cancer therapies.

The working mechanism of the ChemoTAP system in enhancing tumor cell killing is as follows. By inputting intensive SAW pulses directly onto cells, ChemoTAP significantly increases cell membrane permeability, enhancing cisplatin uptake and improving the chemotherapeutic efficiency. Moreover, ChemoTAP also activates ROS-mediated apoptosis signaling pathways during the pulse stimulation process. This study provides a possibility of integrating acoustofluidics technologies with conventional chemotherapy protocols, offering a novel strategy to improve treatment outcomes. Moving forward, continued optimization of ChemoTAP parameters and further evaluation of clinical applicability will be essential for advancing ChemoTAP toward *in vivo* mechano-chemotherapy applications and broader adoption in precision oncology.

While the ChemoTAP system is effective, reliable, and fully compatible with benchtop protocols, several areas warrant further investigation to maximize its therapeutic potential. Expanding ChemoTAP stimulation to a broader range of mechanically distinct cell types will be essential for determining its versatility and optimizing treatment parameters across different tumor models. Additionally, a deeper understanding of the trade-offs between ChemoTAP stimulation parameters, including SAW frequency, amplitude, and pulse duration, could further refine its efficacy. Currently, ChemoTAP operates at a fixed SAW frequency, but its modular design enables the integration of variable-frequency acoustic transducers,^72,73^ allowing for dynamic tuning of mechanical stimulation to explore diverse mechanotransduction pathways in oncology. Although the ChemoTAP system demonstrates solid results in Petri dish-based SAW stimulation, it remains an *in vitro* approach. The proposed system will face several challenges for *in vivo* cancer cell killing applications: high-frequency SAWs exhibit limited tissue penetration, restricting applicability to superficial tumors; *in vivo* biological complexity (*e.g.*, vascularization, tissue heterogeneity) may attenuate acoustic energy and alter mechanical stimulation responses; and challenges in acoustic coupling between the device and tissue will further constrain utility. Nevertheless, the current work provides valuable insights for *in vivo* experimentation, such as SAW duration and input power selection. These advancements lay the groundwork for further development. Future work should focus on validating SAW penetration capability, performing acoustic field intensity attenuation correction in tumor models, and developing targeted drug carriers to broaden the feasibility of the ChemoTAP.

In conclusion, the combination of ChemoTAP stimulation and cisplatin supports the hypothesis that mechanosensitive ion channel activation by acoustics-induced mechanical forces improves the *in vitro* efficacy of synergistic mechano-chemo tumor cell killing at the cellular level. Beyond its contribution to synergistic work, the ChemoTAP also acts as a non-invasive, tunable, and effective mechanical stimulation tool, activating ROS-mediated apoptosis signaling pathways. This study offers key insights into the broader biological implications of acoustic stimulation, highlighting its potential for advancing targeted drug delivery, overcoming chemotherapy resistance, and enabling next-generation mechano-chemotherapy strategies. These findings reinforce the promise of ChemoTAP as a transformative tool for therapeutic innovation in oncology.

## Author contributions

R. Z., K. L., K. Y., and T. J. H. designed the research. R. Z. and K. J. fabricated devices. R. Z., K. L., K. Y., Y. C., and M. L. conducted experiments, analyzed the data, and prepared the manuscript. R. Z., Q. W., X. X., and J. X. conducted the numerical simulations. J. D. H. M., J. R., N. U., and S. Y. helped with the manuscript editing. T. J. H. and Y. C. supervised the work and edited the manuscript.

## Conflicts of interest

T. J. H. has co-founded a start-up company, Ascent Bio-Nano Technologies Inc., to commercialize technologies involving acoustofluidics and acoustic tweezers.

## Supplementary Material

LC-025-D5LC00419E-s001

LC-025-D5LC00419E-s002

## Data Availability

Supplementary information is available. See DOI: https://doi.org/10.1039/D5LC00419E. All data supporting the findings of this study are available within the article and its SI files. Additional raw data and the custom MATLAB script used for fluorescence intensity analysis are available from the corresponding author upon reasonable request.
