# Supplementary material for: Enhancing cancer therapy via acoustics: chemotherapy-enhanced tunable acoustofluidic permeabilization (ChemoTAP)
Source: Lab Chip. 2025 Oct 21;25(23):6314–23. doi: 10.1039/d5lc00419e (PMC12538558; doi:10.1039/d5lc00419e)
Supplement: LC-025-D5LC00419E-s001 [file LC-025-D5LC00419E-s001.pdf]

Supplementary Information (SI) for Lab on a Chip.
This journal is © The Royal Society of Chemistry 2025

Supplementary Information (SI) for Lab on a Chip.
This journal is © The Royal Society of Chemistry 2025

Enhancing cancer therapy via acoustics: Chemotherapy-enhanced tunable acoustofluidic permeabilization (ChemoTAP)

Ruoyu Zhong,[a†] Ke Li,[a†] Kaichun Yang,[a] Qian Wu,[a] John D.H. Mai,[b] Joseph Rich,[c] Ying Chen,[a] Xianchen Xu,[a] Jianping Xia,[a] Neil Upreti,[c] Ke Jin,[a] Shujie Yang,[d] Mingyuan Liu,[e] and Tony Jun Huang [a*]

[a] Thomas Lord Department of Mechanical Engineering and Materials Science, Duke University, Durham, NC 27708, USA. E-mail: tony.huang@duke.edu
[b] Alfred E. Mann Department of Biomedical Engineering, University of Southern California, Los Angeles, CA, 90089, USA.
[c] Department of Biomedical Engineering, Duke University, Durham, NC, 27708, USA.
[d] Department of Mechanical Engineering and Applied Mechanics, University of Pennsylvania, Philadelphia, PA, 19104, USA.
[e] Department of Electrical and Computer Engineering, Duke University, Durham, NC 27708, USA.
[†] These authors contributed equally to this work.

Supplemental Results

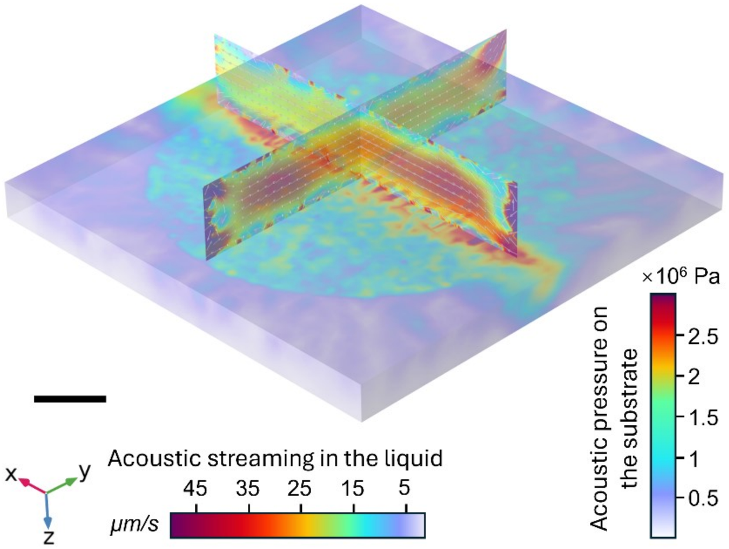

Fig. S1 3D simulation of the ChemoTAP system. Three-dimensional simulation of the acoustic pressure distribution on the $LiNbO_3$ substrate and the acoustic streaming within the liquid in the Petri dish. Arrows in gray color represent the direction of acoustic streaming. A weak streaming is observed in the simulation plot. Scale bar: 1 mm.

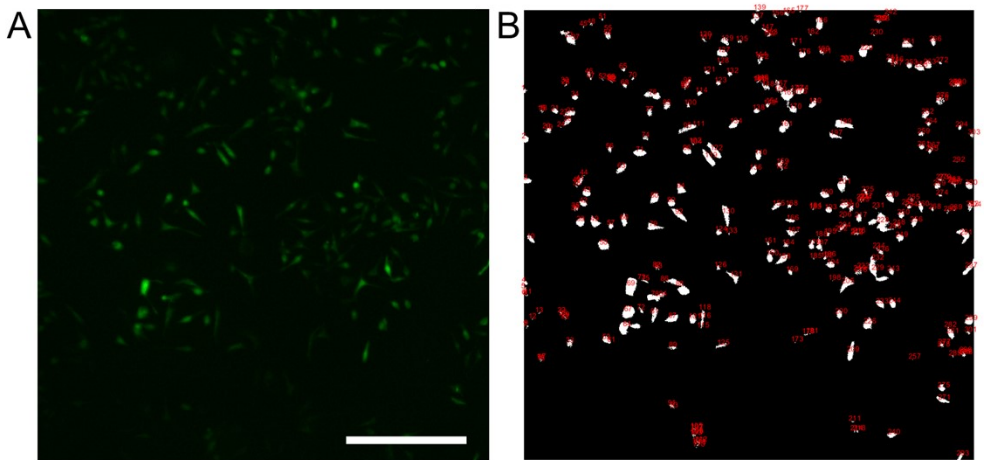

Fig. S2 Automated cell labeling for fluorescence intensity tracking. (A) Fluorescent cells were identified and numbered using (B) a homemade program, enabling the automatic collection of single-cell fluorescence intensity variations over time. Scale bar: 400 µm.

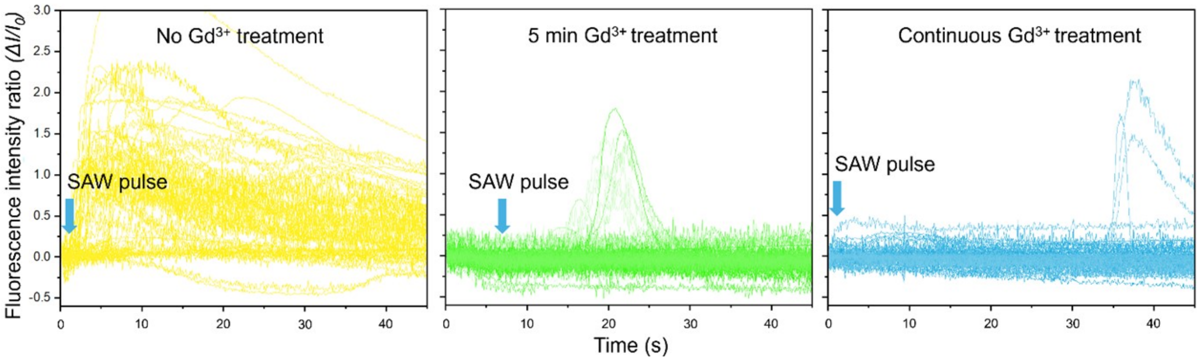

Fig. S3 Intracellular calcium ion flux intensity in single HeLa cells with different Gd^{3+} treatments. The curves of single HeLa cell fluorescence intensities in the 'no Gd^{3+} treatment' group exhibit strong and rapid increases following SAW pulse stimulation. In contrast, the '5 min Gd^{3+} treatment' group shows minimal response to SAW stimulation in almost all cells. Similarly, in the 'Continuous Gd^{3+} treatment' group, this inhibition of cell response is pronounced, with only about three cells demonstrating a delayed uptake of intracellular calcium ion flux. (n: No Gd^{3+} = 88; 5 min Gd^{3+} = 347; continuous Gd^{3+} = 118).

Note S1. MATLAB Script for tracking and recording the fluorescence intensity changes of each single cell in the field

This MATLAB script is designed to track and record the fluorescence intensity of individual cells over time. The script requires MATLAB 2017b or later to run. To execute the analysis, users should run the main file ‘wroks.m’. The input data should be provided as a video file (e.g., ‘avi’), and the results will be saved in a table format as ‘Excel.txt’. For additional details, the source code of the main file ‘works.m’ is provided below:

```
clc;clear;
video = VideoReader('XX.avi'); %input video/source data
Il = read(video,1); %locate the position of every cell
img1=rgb2gray(Il); %process the image
img11=imbinarize(img1);
img2=bwareaopen(img11,20);%remove small regions
img3 = bwconncomp(img2,8)
edgeimg=edge(img2,'Canny',0.5);
doubel_img3_cell=im2double(img2);
doubel_img3_edge=im2double(edgeimg);
Img3=labeloverlay(doubel_img3_cell,doubel_img3_edge);
labeledImage = bwlabel(img2); %label the cells being recorded
Num_obj = max(labeledImage(:));
hold on;
status=regionprops(img2,'BoundingBox');
centroid = regionprops(img2,'Centroid');
area = regionprops(img2,'Area');
figure();
imshow(img2);

for i=1:Num_obj;
   %Number mark
   text(centroid(i,1).Centroid(1,1)-15,centroid(i,1).Centroid(1,2)-15, num2str(i),'Color', 'r');
end
hold off
Number=img3.NumObjects;
Cells=img3.PixelIdxList;

% recording cell fluorescence intensities
frames=video.NumberOfFrames;
Excel=zeros(frames,Number);
for i = 1:frames;
   imagei=read(video,i);
   imagei=rgb2gray(imagei);
   for j =1:Number;
      Onecell=cell2mat(Cells(j));
      G=0;
      for k=1:size(Onecell);
```

```
            G=G+double(imagei(Onecell(k)));
        end
        grayvalue=G./size(Onecell);
        Excel(i,j)=grayvalue(1);
    end

end
```
